# The creation and characterisation of a National Compound Collection: the Royal Society of Chemistry pilot†
†The authors declare that this work was in part funded by the Royal Society of Chemistry.
‡
‡Electronic supplementary information (ESI) available: Detailed acknowledgement of contributions. Script written by NQuiX for evaluation of structural uniqueness. See DOI: 10.1039/c6sc00264a


**DOI:** 10.1039/c6sc00264a

**Published:** 2016-02-23

**Authors:** David M. Andrews, Laura M. Broad, Paul J. Edwards, David N. A. Fox, Timothy Gallagher, Stephen L. Garland, Richard Kidd, Joseph B. Sweeney

**Affiliations:** a Royal Society of Chemistry , Thomas Graham House, Science Park, Milton Road , Cambridge , CB4 0WF , UK . Email: david.andrews@astrazeneca.com; b School of Chemistry , University of Bristol , Bristol , BS8 1TS , UK; c Scicate Limited , Mendip Court , Bath Road , Wells , Somerset BA5 3DG , UK; d NQuiX Ltd , Causeway House, Dane Street , Bishops Stortford , Hertfordshire CM23 3BT , UK; e Department of Chemical Sciences , University of Huddersfield , Huddersfield HD1 3DH , UK

## Abstract

We present a summary of the National Compound Collection (NCC) pilot; which harvested chemical structure data from 746 publicly-available PhD theses to create an enhanced database of diverse and interesting (largely organic) molecular entities. The database comprised ∼75 000 structure entries, of which 70% were new to ChemSpider at the time of upload. The dataset was evaluated for structural uniqueness by twelve external drug discovery groups from the pharmaceutical, biotech, academic and not-for-profit sectors. These partners generated data reported here comparing the NCC pilot with their in-house compound collections. The proportion of NCC structures considered to be useful for drug discovery ranged from 5–80% depending on the strictness of the filters used; most interestingly from a drug discovery standpoint ∼13k NCC compounds (18% of the NCC) passed the filters and were of good diversity. These compounds are quite different from those that are already present in the screening collections but not so different that they are no longer considered to be drug-like. In general, the drug discovery teams would consider these compounds to be high value molecules for inclusion in their screening collections. This pilot addressed the potential value of unpublished data and explored the practicalities of large-scale data extraction, to inform both retrospective and prospective extraction of chemical data from theses.

## Abstract 2

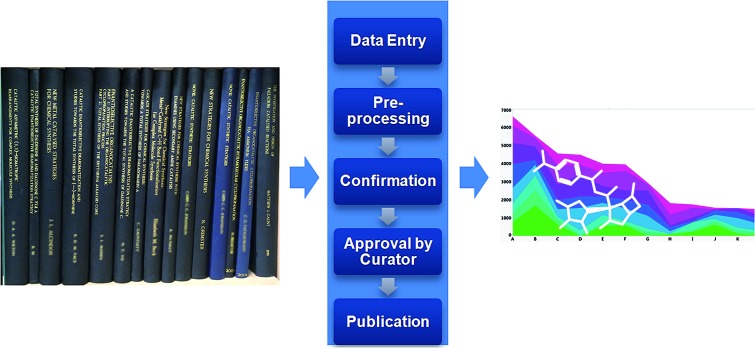
We report the extraction of compound data from historical literature, making it chemically searchable. Evaluation by drug discovery groups reveals the utility of this approach.

## Introduction

Arguably the most important output of UK chemistry departments is the cohort of PhD students that have been trained in research methods and experimental techniques; it has been estimated that more than $20Bn has been contributed to the global pharmaceutical sector by UK-funded/based PhDs.^1^ All of those students produce a thesis and many of those theses contain new chemical entities or new (and often better) ways to synthesise important chemical entities, with a strict requirement of the degree being that compounds are adequately and appropriately characterised. While a good deal of a PhD student's work is published, any practicing academic knows that there is often also a significant body of results within a thesis that, because they didn't deliver “the research goal”, remain unpublished in the primary literature. This body of unpublished “big data” frequently includes novel compounds, experimental data which will also meet the degree requirements of quality and characterisation. These inaccessible and not computer-searchable “hidden data” represent a valuable and untapped resource for chemists and indeed the wider molecule-using research community.

It is also important to appreciate that the process of a PhD degree necessarily results in the publication of the thesis, so theses are free-standing scientific documents in their own right, which are available, possibly subject to a period of embargo and with varying accessibility, once the degree requirements have been met and the final copy has been submitted.

We have carried out a pilot study, the goal of which was to evaluate the potential and realise the value of these hidden data by harvesting chemical structure information from PhD theses to create an enhanced database of novel and interesting molecular entities. It was important that the theses made available were published (and consequently within the public domain) and not subject to IP issues, in that journal publications and any patent claims had already been made. It was also apparent that an ideal vehicle for structural data deposition, dissemination and retrieval was the Royal Society of Chemistry (RSC)'s ChemSpider,^2^ already a validated chemical structure database. We envisaged such a database also as being able to (i) benefit from harvesting a wealth of “legacy” data – *i.e.* from many older theses – and (ii) provide a means to capture new theses as these are produced/published going into the future. The latter would then provide a mechanism for constant refreshing and extending a wide-ranging and comprehensive “tangible” database with new structural entities. In this context, a tangible database is a database of compounds which are known to have been synthesised; by comparison a “virtual” database contains the structures of compounds which *could* be made (*cf.* Hann^3^). In contrast, a “physical” collection refers to a situation in which compounds exist as physical samples available for screening. While a tangible collection has significant advantages over virtual libraries, it is widely recognised that eventually, access to a physical sample is required in order to confirm activity and initiate a follow-up programme. Since the completion of this work, Research Councils UK (RCUK), on behalf of the UK Open Research Data Forum, published a draft Concordat on Open Research Data, which sets out both principles and expectations of good practice in publishing research data openly^4^ (see Box 1). These principles are in good alignment to many of the issues and opportunities we identified during the course of this pilot.

Box 1: the ten concordat principles

(1) Open access to research data is an enabler of high quality research, a facilitator of innovation and safeguards good research practice.

(2) Good data management is fundamental to all stages of the research process and should be established at the outset.

(3) Data must be curated so that they are accessible, discoverable and useable.

(4) Open access to research data carries a significant cost, which should be respected by all parties.

(5) There are sound reasons why the openness of research data may need to be restricted but any restrictions must be justified and justifiable.

(6) The right of the creators of research data to reasonable first use is recognised.

(7) Use of others' data should always conform to legal, ethical and regulatory frameworks including appropriate acknowledgement.

(8) Data supporting publications should be accessible by the publication date and should be in a citeable form.

(9) Support for the development of appropriate data skills is recognised as a responsibility for all stakeholders.

(10) Regular reviews of progress towards open access to research data should be undertaken.

We also saw, given the rapidly increasing power of *in silico* tools, that there was an exciting opportunity to filter a structural database in order to prioritise and select a subset of molecules that could then be targeted for re-synthesis. Ultimately, this could provide a starting point for a separate physical National Compound Collection aligned to an *in silico* tangible collection, the pilot study for which is described here.

We envisaged any database (be it tangible or physical) as a widely applicable resource that should be of use to any sector of “molecule users”, where in particular the tangible component (*i.e.* the *in silico* element) could be triaged/filtered in a bespoke manner or not at all according to the demands associated with a particular end-user.^5^

We also recognised that there was a variety of constituencies that needed to engage to enable this project to establish the credibility and momentum necessary for sustained success. Stakeholders included “producers” (academic chemistry groupings involved in synthesis), end-users (both academic and industry, from SMEs to multinationals and across various end-user sectors), and associate industry players (*e.g.* companies involved in contract synthesis, development or use of new *in silico* tools, collection and curation of substantial collections). The support of research funders was also seen as important since EPSRC funds some 40% of UK chemistry PhDs and EPSRC, BBSRC, MRC and the Wellcome Trust all fund a wide range of “molecule users”. Gaining input from academic institutions was seen as essential since they are the primary source of structural data and often Intellectual Property owners. Finally, professional and other bodies (*e.g.* RSC, British Library) are able to bring various other critical elements to the project. This includes a key role (*e.g.* for RSC) in coordinating the engagement of these various “interested parties”.

## Goals of the pilot

### Goal 1: to explore the processes and procedures required to extract structural data from academic theses, to inform a future national scale activity

We used a manual data extraction approach for the pilot study, following initial experimentation with the use of chemistry intelligent Optical Character Recognition (OCR) software.^6^ Challenges around accessibility of digital copies copyright (we exercised caution in this regard) together with the necessary costs of licences, training and quality assurance of the accuracy of the data collected suggested that a national scale activity would require industry standard mixed approaches already employed by commercial database providers. Work carried out by the British Library examined the accessibility of the theses across institutions, in terms of availability of digital copies and the licensing of theses by different institutions. The implications of the recent copyright exemption for text and data mining in the context of the project were also used to inform this pilot and the EThOS (e-theses Online Service ; http://ethos.bl.uk/About.do) digital collection.

### Goal 2: to demonstrate an ability to collect and collate a database of structural and bibliographic information within RSC's ChemSpider

ChemSpider is a free-to-access resource developed with RSC that is well-suited for hosting data of the type we would deposit, especially as a demonstrator for the pilot. Modifications were required to provide a specialist deposition interface to include thesis-specific details. Deposition and export tools provided an easy method for the data collectors to remotely update and publish the collected data. The pilot data cleared for release are available through ChemSpider, and as a Creative Commons CC0 licensed download. The CC0 licence has been applied to this data in line with the principles applied to linked data by BioMed Central^7^ and Nature Publishing Group.^8^,^9^

### Goal 3: to demonstrate the searchable nature of this tangible database using customised filters and specifically to exploit the database *via in silico* screening against a series of “societally important” proteins

Linked to this was the parallel use of the similarly-sized French-based compound collection.^10^ The latter is an academically-derived physical collection that has been screened for bioscience application. We applied parallel *in silico* screening of the two collections to a series of protein structures using the Bristol University Docking Engine (BUDE).^11^ The French collection provided us with the opportunity to source, screen and validate the principles and methodology of the *in silico* screening and served, consequently, as a proxy for a “physical” equivalent to our pilot data collection. The outcome of this part of the study, which involved *in silico* assessment and then physical screening using the relevant French hits against some 30 different protein targets, will be the subject of a separate publication and will not be covered in any more depth here.

### Goal 4: assessment by external bioscience/pharma partners to evaluate the “uniqueness” and relevant “structural space” coverage of the pilot collection *vs.* their own in-house collections

Our initial plan was to apply filters to select for compounds to target for re-synthesis, but it was clear following consultations that it is more important to allow any end-user to filter according to need, so we chose not to apply any filters to the tangible collection.

The objective in this aspect of the pilot was to filter and profile the collection against collections assembled and maintained externally *vs.* institution-specific interpretations of molecule quality. We anticipated that the comparison against SME and larger company databases across the agrochemical and pharmaceutical space would be much more informative than a snapshot against a single company.

## Methods

We engaged 15 university Chemistry departments to provide representative theses: Bradford, Bristol, Bath, Cambridge, Cardiff, Glasgow, Huddersfield, Imperial, Leeds, Leicester, Loughborough, Nottingham, Oxford, Southampton, Strathclyde and UCL. Where feasible, we divided these into geographical clusters (*e.g.* Nottingham/Loughborough/Leicester) with a lead institution (and identified lead academic) within which we planned to locate one or more “data collectors”; clusters of three universities had two data collectors assigned. In addition, St Andrews and Birmingham, *via* the British Library, provided us with access to their digital repository and we also selected theses from these universities.

Our data collectors were recruited within the lead university and were current or newly graduated chemistry PhDs. Their brief was to cover their local or cluster of universities and to target as wide a variety of different synthetic theses as possible. We set up and ran a training and briefing session in February 2014 and the cohort rapidly established a blog site that enabled them to deal with unforeseen issues collectively and share experiences and good practice. The data collectors worked to a common protocol and were managed *via* weekly Skype conferences or one-to-one meetings. There was an initial period associated with “learning the processes” but all data collectors quickly reached very productive levels.

We sought to include a variety of theses covering different topics to try to maximise diversity of structure, and we only used published (*i.e.* openly accessible and non-restricted) theses. To ensure that contributors could make best use of the theses available to select from, we undertook to ask permission for final release of the complete data collection.

**Fig. 1:**
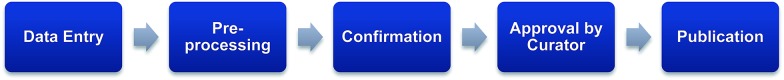
Data entry comprised deposition of thesis information plus structure information and metadata concerning compound characterisation data plus thesis location. Pre-processing provided an opportunity for the submitter to check summary information. There was an opportunity to upload ESI (*e.g.* Pubmed ID or DOI) at the confirmation stage; it was also the final opportunity for the Submitter to check and amend the uploaded information. Prior to final publication to the embargoed NCC pilot collection, each entry was approved by an RSC data curator.

Input forms were provided to key in the thesis level information, and then individual structures were pasted in from ChemDraw together with accompanying compound identifiers and data availability flags (see Fig. 1). Once data deposition was complete (based on InChI), ChemSpider automatically calculated and added a range of additional properties (*e.g.* SMILES string, mol. wt., mol. formula, p*K*_a_, *c* log *P etc.*) that provided the basis of a future filtering mechanism. The entry also included indexing data summarising the analytical characterisation carried out and reported in the thesis, as well as information around chirality. A key data entry within ChemSpider was the bibliographic details of the thesis: *e.g.* author, supervisor, publication year, thesis title, university.

During Feb–June 2014, we collected 45 098 individual structure entries (as above). Duplicates were removed and where we had an entry for a racemic compound, the individual enantiomers were generated as unique entries for analysis on output. Where we had a single enantiomer (or a structure with up to two undefined stereocentres), RSC also generated the “other” enantiomer and/or diastereoisomers. This step was taken to maximise the chances of a calculated match in the *in silico* screening against protein targets (Goal 3).^12^

**Fig. 2:**
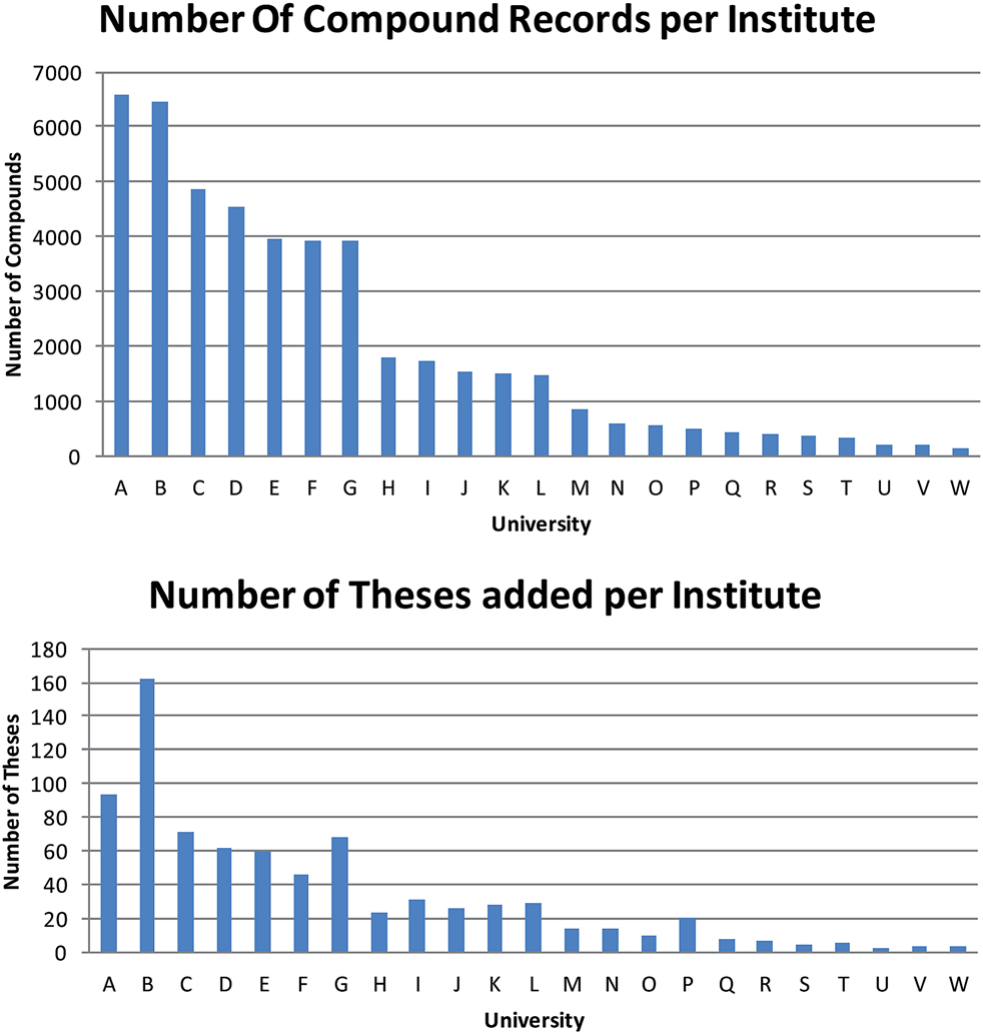
Number of compounds abstracted per institute compared to the number of theses indexed per institute (institute ordering is the same).

There was a notable variation in the number of theses obtained from each institute (Fig. 2). Quantifying this variation is further complicated by the fact that the university recorded in ChemSpider is that of the current or last known institute of the PhD supervisor (and thus not necessarily the university that awarded the PhD degree). In turn, this means that the number of institutes recorded in Fig. 2 is greater than the 15 that formally partnered this pilot study. Finally, this leads to a considerable variation in the number of compounds abstracted from each university and there is little correlation between the number of theses extracted and the number of compound records obtained from each department.

At the conclusion of the data entry phase, a total of 45 098 individual chemical structures had been input and expanded to ∼75 000 unique structure entries on output. Of the 45 098 data entries, ∼31k (∼70%) were new structural entities to ChemSpider.

## Thesis mining

In parallel to identifying the 15 universities to supply the theses, and working out the practical details previously described, there were extensive discussions between the British Library and the pilot study group, focussing on discoverability, accessibility and licensing issues, as well as the impact on copyright of text and data mining activities.

Some universities have already undertaken large scale scanning of PhD theses to convert paper to an electronic format, but if this type of resource is to have real value, the level of metadata available that can be used to retrieve more detailed information needs to be significantly improved in many cases. This reflects on another issue, which is the level of consistency between different institutions in terms of how they collate, curate and offer for dissemination thesis data and metadata, as well as different practices in terms of the licensing and subsequent availability of the theses. Currently often only minimal searchable data are associated with a paper thesis: student name, title, date. Perhaps department name is included in a catalogue but as a rule, theses are not searchable by supervisor nor can entries be interrogated by a standard set of keywords (subject/discipline area and sub-discipline and other key words *etc.*). If this issue around metadata is not addressed, then the prospect of efficiently and cost-effectively retrieving data (structural data in our case) from legacy theses becomes almost insurmountable.^13^ Given that the development of electronic research archives within universities is already underway, a recognition of and response to this issue of inadequate metadata is a matter of urgency. The risk otherwise is that the electronic variants suffer from the same issues of inaccessibility (and hurdles to data retrieval) as the current physical thesis collections.

At the outset, copyright was viewed as another significant issue in this area, however there have been some pertinent changes to UK copyright law in 2014 and it is now feasible to apply automated tools to extract data without the need for permissions to be gained, provided that this is done for a non-commercial use.^14^ The guidance published by the UK Intellectual Property Office provides a clearer understanding of the relationship between copyright, automated data extraction and commercial exploitation. Having a much-improved understanding of these issues, we were able to tackle Goal 4 – assessment by external bioscience/pharma partners to evaluate the “uniqueness” and relevant “structural space” coverage of the pilot collection.

## Results

The National Compound Collection (NCC) dataset was evaluated for structural uniqueness *via* a custom-developed script written by NQuiX (see ESI‡). A number of drug discovery groups from the pharmaceutical, biotech, academic and not-for-profit/charity sectors were provided with both the dataset and the program.

**Fig. 3:**
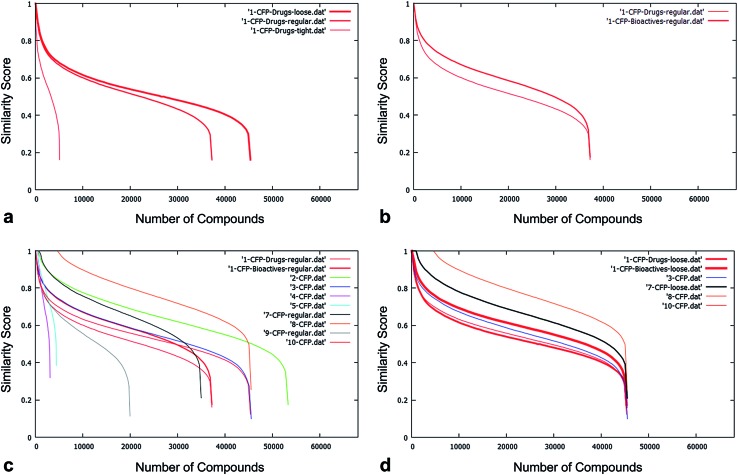
(a) The effect of tighter filtering on comparison of the NCC pilot with a standardised set. As expected, when the loosest criteria are applied, the greatest number of compounds pass the filters and the curve plateau is at around 45 000 compounds; the application of the tightest filtering results in around a plateau at around 5000 filter passes. (b) The effect of comparing the NCC pilot output to collections of increasing diversity. The standardised database of Bioactives is both larger and more chemically diverse than the Drugs database; therefore NCC compounds have a higher probability of being scored similar to a compound in the Bioactives database than in the Drugs database – hence the area under the curve is greater for the comparison to Bioactives. (c) The comparison of the NCC to the nine companies' corporate collections. Curves plateau at different levels, reflective of different definitions of filter stringency for “Normal Filtering”. Areas under the curve vary according to the size and diversity of each organisation's collection. (d) An example of four organisations that have implemented filters of very similar stringency. The differing area under the curve reflects each organisation's different size and diversity of corporate collection. In this example, all would benefit, but the pilot NCC would add value to the diversity of collections in the order 10 > 3 > 7 > 8.

Of these, 9 partners (AZ, Domainex, Dundee, Evotec, GSK, MRC-T, Pfizer, Syngenta and UCB) generated data comparing the NCC with their in-house compound collections; NQuiX provided extensive data for the NCC *versus* marketed drugs, bioactive compounds from the literature and commercially available “purchasable” samples (Fig. 3); and both Lilly and the Structural Genomics Consortium provided feedback on the value of the dataset using their own methods.

The standardised comparison process measured the novelty of the compounds in four different ways: using two different types of chemical fingerprint (Chemical Hashed Fingerprint (CFP)^15^ and Extended Connectivity Fingerprint (ECFP)^16^), counting the number of novel Bemis–Murcko structural frameworks,^17^ and counting the number of novel ring systems. The software used for this work provided a ready means to fragment molecules into constituent parts but required a rule set to be defined for the relevant transformations. Using a simple breakage of bonds between ring atoms and non-ring atoms does not produce what could be intuitively regarded as the relevant “ring systems”. Such a procedure would generate a contiguous group of ring atoms for more complex fused and joined rings such as steroid frameworks and biaryls but it would also cleave the carbonyl oxygen from a lactam, for example. In keeping with a number of other groups,^18^–^20^ we felt that atoms alpha to the ring system should be retained. However, rather than dealing only with the specific issue of doubly bonded pendant atoms, we wanted to know the full substitution pattern around rings since, if novel, these were suggestive of new options for decoration – and hence chemical diversity – even if the ring core had itself previously been described. We were less concerned to know about variation in atom type at the substitution point, so converted them to a generic atom (denoted “A” for any). In summary, the advantage of including the attachment point into the “ring system” as defined is that this can make otherwise equivalent rings appear very different in terms of important characteristics such as synthetic route, scope for expansion (*i.e.* diversity) and fit to target.

Whilst the NCC dataset contained just over 75 000 entries in total, this figure was reduced to ∼68 000 following structure regularisation^21^ and elimination of, for example, isotopically labelled variants and alternative salt forms. The number of compounds could (if desired) be further reduced by the application of sub-structural and property filters that seek to ensure “drug-likeness”.

The principle of attempting to improve drug-likeness for designed molecules in biopharmaceutical science (encompassing aspects which include potency, selectivity, absorption, distribution, metabolism, excretion and toxicity characteristics) is now well established and interpretations of “quality” have been published by multiple groups.^5^,^22^ Each drug discovery group was therefore free to impose its own controls over the filters and was encouraged to use two differing levels: “loose” filtering, appropriate for the identification of potential tool molecules, and “regular” filtering, consistent with more usual Compound Collection Enhancement/Lead Generation type activities.

The proportion of the 68k unique NCC structures passing the drug-likeness filters ranged from approximately 5% to 80% depending on the strictness, yielding between ∼3k and ∼55k compounds for the diversity analysis. On average, ∼50% of the structures passed the filtering process (see Fig. 3).

Typically ∼2k structures (3% of 68k) were found to be very highly diverse compared to the existing collections (<40% Tanimoto similarity of CFP). A fair proportion of these look to be rather small (there is usually no lower bound on MW in the filters) and some may be appropriate for fragment-based drug discovery efforts. Other compounds within this subset looked potentially undesirable, for example, for reasons related to metabolic stability. These structures have most likely passed the filtering process because they represent new chemotypes but, being previously unseen, it is simply that no sub-structural drug-likeness filter has been generated for them as yet. This is very much in line with guidance issued by Lilly in their PD^2^ initiative – compounds that are too dissimilar to their collection cease to be drug-like and slip through the filters and are discarded.^23^,^24^

**Fig. 4:**
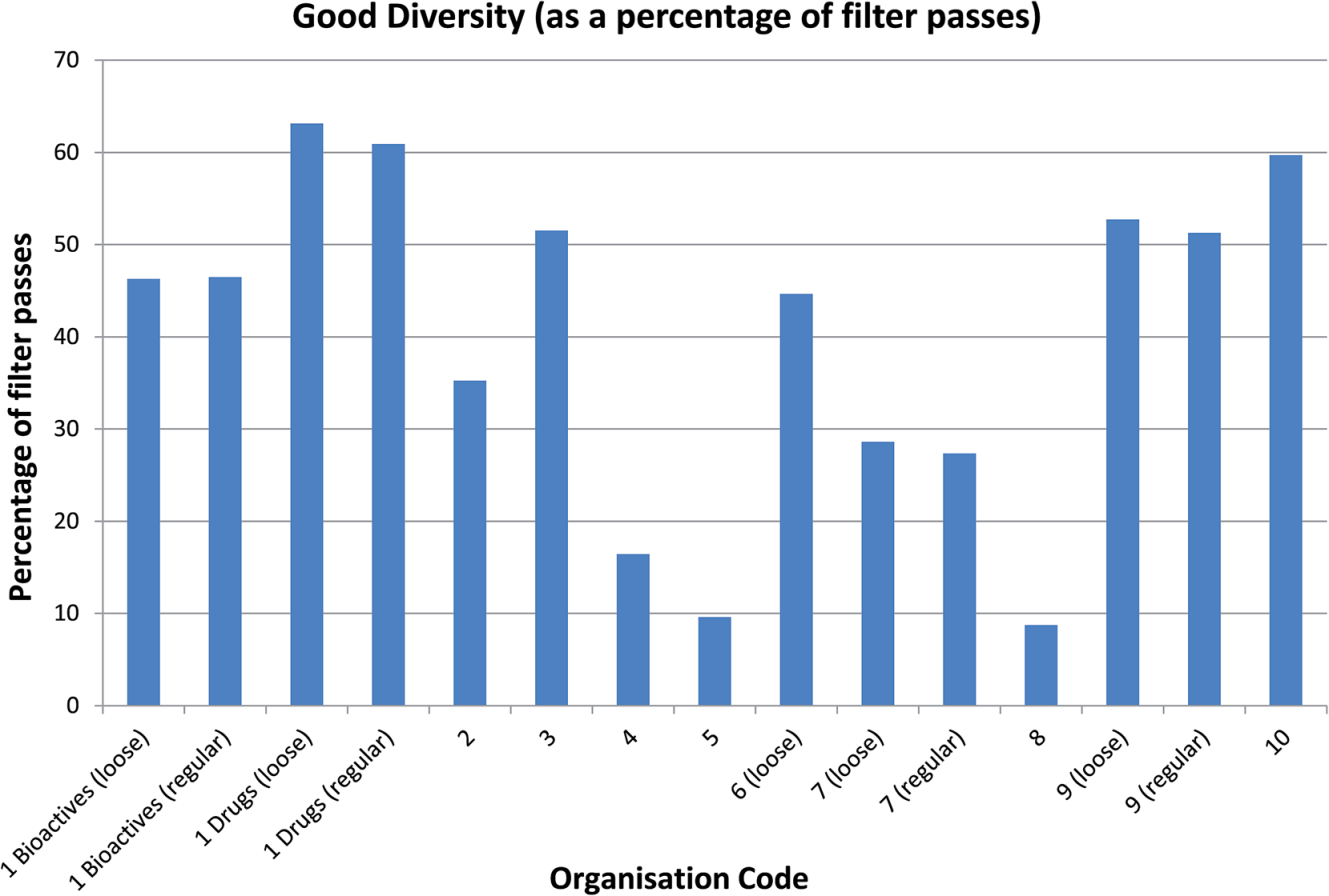
Good diversity compounds identified by each company as a percentage of each company's filter passes. Companies 4 and 5 have a very high filtering stringency, and we suspect that their passes are also much smaller in molecular size. The lower molecular size and complexity makes it harder for the filter passes to be diverse relative to known compounds. With company 8, the filtering stringency is about average but they have a collection with greater similarity to the NCC and hence fewer molecules with good diversity. Interestingly, by this measure, the similarity of the comparator set to the NCC is more important than the stringency of definition. Note that comparing loose or regular filtering yields very similar numbers of good diversity compounds as a percentage of filter passes. In this example, the smallest comparison set is the Drugs file; unsurprisingly, over 60% of compounds are of good diversity as a percentage of filter passes. Aside from the already mentioned companies 4, 5 and 8, for the majority of companies, 30–50% of compounds pass filters and demonstrate good diversity. This sweet spot of diverse, filter-pass compounds represents around 18% of the NCC compounds.

Arguably more interesting from a drug discovery standpoint are the ∼13k compounds with good diversity (40–60% Tanimoto similarity of CFP). These compounds – representing around 18% of the NCC pilot – are quite different from those that are already present in the screening collections but not so different that they cease to be drug-like. Given that the database contains chemistry in its broadest sense and captures reagents as well as final products, this is very encouraging and many drug discovery teams would consider these compounds to be high value molecules for inclusion in their screening collections (Fig. 4).

**Fig. 5:**
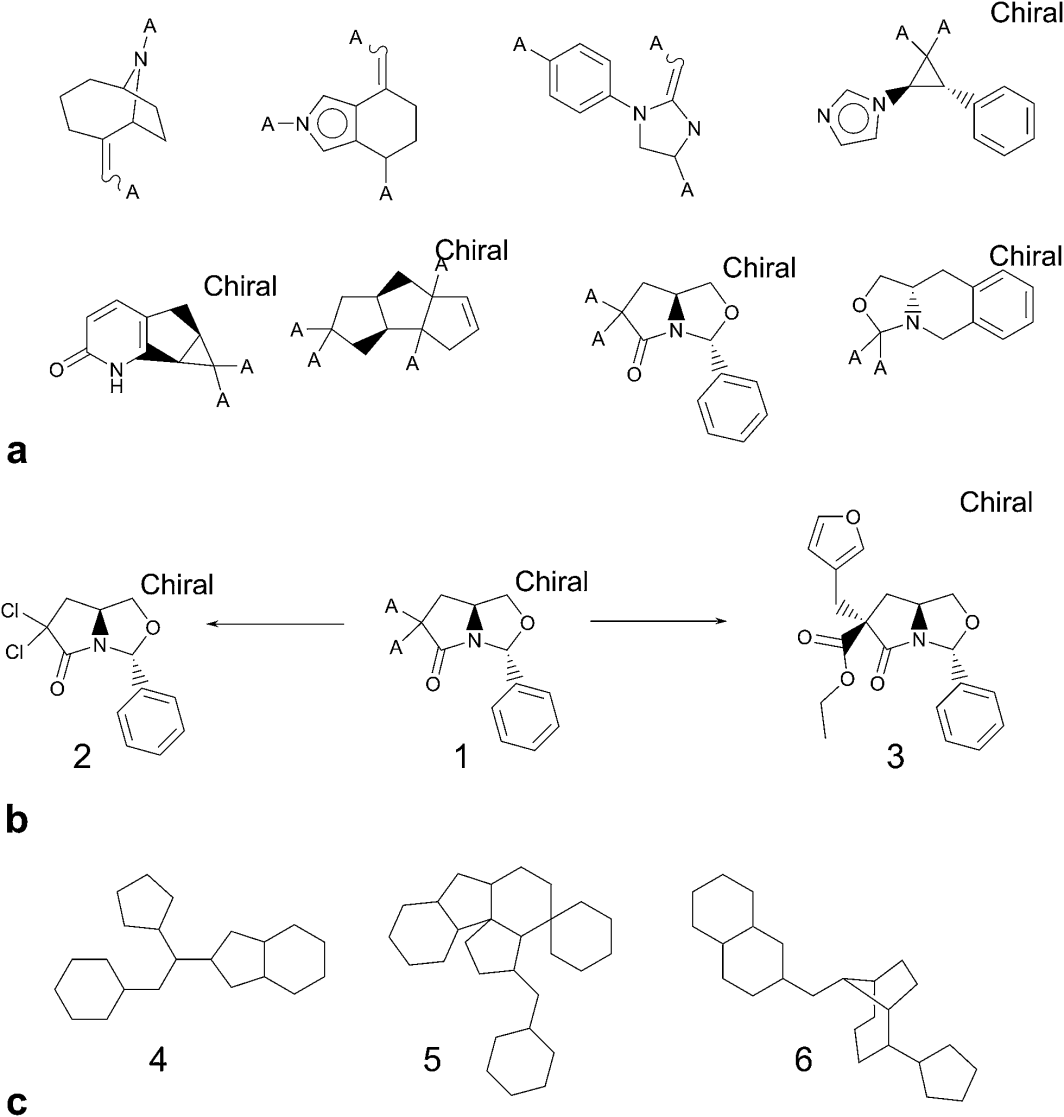
(a) Illustrative examples of novel ring systems relative to known bioactive compounds. (b) Ring system **1** corresponds to two unique NCC entries **2** and **3**. Compound **2** possibly lacks significant scope for decoration; however many users would consider compound **3** to be more desirable. (c) Examples of frameworks that are novel compared to known bioactive compounds. **4** is quite simple and we were surprised that it was classified as novel; **5** is an example at the opposite end of the complexity spectrum and probably undesirable to many; **6** is another interesting framework with plenty of scope for elaboration.

Focussing on rings and frameworks as a methodology for analysis highlights significant potential diversity in the drug-like subset of NCC. In particular, the selection is highly diverse, with only a very small number of analogues per chemotype. On average, there were 6726 distinct ring systems of which only 861 (∼13%) were already present in screening collections and 2065 distinct frameworks of which only 742 (∼36%) were precedented (Fig. 5).

In the foregoing discussion, the results slightly overstate the novelty of the NCC in comparison to corporate collections. Some ring systems occur in the NCC that are unsubstituted and low in molecular weight; *i.e.* they are the complete structure, not a substructure and are therefore classified as reagents. For many organisations, the corporate compound registry would contain reagents but these would not be part of the screening collection due to their reactivity, scope for assay interference and undesirability as screening hits. We asked our partner companies to generate comparison data against their screening collections and so the lower molecular weight entities are less likely to be present unless represented as part of a fragment-based set. This finding is also reflective of the fact that our data collectors were encouraged to upload all of the examples from a thesis' experimental section, since at the point of upload, they were in no position to reliably judge what is novel and what is not. To estimate the overstatement of novelty, the pilot NCC was compared to the control Bioactives database, using a regular level of filtering, and 668 from 6385 “novel” ring systems are unsubstituted. Extrapolation across the data set would suggest an overstating of novelty of around 10%.

The small number of compounds representing each chemotype (as well as the fragment-sized scaffolds) presents opportunities for academic and commercial data exploitation. As an example, computational tools are freely available that can prospectively focus efforts on those scaffolds that have the potential to target novel lead-like chemical space. Recently, Nelson and Marsden have developed and launched LLAMA (http://llama.leeds.ac.uk), an open-access tool that allows the lead-likeness of scaffolds to be assessed. In most cases, the originators of the thesis chemistry described best understand the opportunities and weaknesses in the chemistry described therein; hence they are well placed to interrogate their own chemistry with design tools such as LLAMA, planning follow-on chemistry that fills some of the currently sparsely-populated chemical space. An example would be the potential use of the NCC in helping to enable synthesis of compounds designed and evaluated through LLAMA (through close neighbours that reside within NCC) either to provide insight into synthetic routes or by diversion of an original idea to a series that has already been enabled in NCC. Learning from the Joint European Compound Library initiative, academic/Contract Research Organisation partnerships are a very efficient way of scaling out this type of diversity-oriented synthesis.^25^

## Future outlook

On the conclusion of data evaluation, each source university has been supplied with a file comprising “their” thesis entries and links to compounds to enable them to take a decision on whether to make their compounds available in the NCC data release. In consequence, of the 45 098 pilot compounds abstracted, 44 430 are available as a CC0 licensed download, as well as available through ChemSpider. Once compounds are released to the public, a “take down” policy will operate so that data are removed if and when a copyright holder objects, which is in line with industry best practice.^26^,^27^

This pilot exercise provided a reference to 45 098 compounds associated with 746 source theses provided by 135 academic supervisors. The analysis carried out with industry partners illustrates that the output has a significant level of novelty compared to both known compounds or to corporate collections. Additionally the analysis *vs.* “quality” filters also illustrates the potential utility of the compounds *vs.* biopharmaceutical applications.

**Fig. 6:**
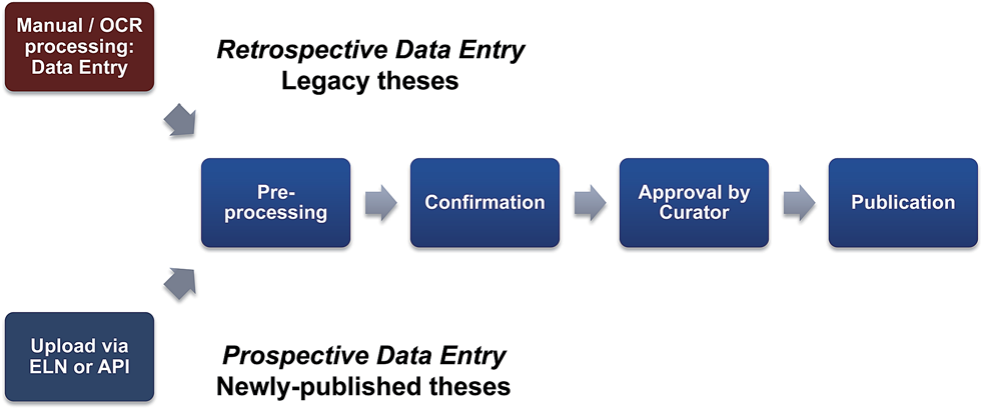
A reappraisal of data entry. A retrospective data entry route will involve a mixture of manual processing and Optical Character Recognition (OCR). The quality and breadth of metadata that are captured will be critical to the success of this approach. Prior to this exercise, much of the metadata captured was at best variable and at worst inadequate. If this is not rectified, the collections created will lack resilience for data mining. Prospective data entry presents different issues. Technically, data entry will be best accomplished using an Electronic Lab Notebook (ELN) or *via* an Application Program Interface (API). Key success factors here will be security and flexibility of embargo which will in turn give research groups the opportunity to record the output of strategic programmes of work across several theses to then allow structure release, once the work is considered sufficiently complete.

Following the successful conclusion of the pilot, the pursuit of a national scale *in silico* collection based on discoverable theses that builds on the results of this pilot study merits serious consideration. In this context, the funding options and mechanisms for longer term sustainability for this activity and a role for UK universities need also to be examined. Given the current emphasis on translational science and assessment of research impact, this is a topic for Research Council consideration and could be seen to be analogous to the block grants announced in 2012 (ref. 28) by RCUK (to fund article processing and open access charges), aiding implementation of RCUK policy on open access to research outputs. A critical component of such an exercise is the development of a clear picture as to the numbers of Chemistry legacy PhD theses available to be extracted, and the cost-benefit ratio involved in mining this likely very significant number of legacy theses; this is a *retrospective deposition* of structural information and a continuation (and scaling out) of this pilot study. Two issues that need to be considered are the fact that many older theses do not have a graphical representation of the structure within the unstructured pdf pages of the experimental section; in this context data extraction from scanned theses could be further compromised if OCR introduces errors into the generation of chemical names. Secondly, while there are large differences in the availability and accessibility of theses across UK institutions, the copyright exception would likely not apply if commercial partners are involved to build sustainability. Taken together, these factors will dictate that a significant level of manual interrogation and intervention will be needed and so in the longer term, the bulk of data may need to be derived from the forward-looking pathway *i.e.* direct data extraction from newly-published theses (Fig. 6). As distinct from the retrospective pathway, this can be termed *prospective deposition* and many of the opportunities and issues for this route have been scoped out in the previously mentioned draft RCUK Concordat.

The National Chemical Database Service (NCDS) is an EPSRC-funded service provided by the Royal Society of Chemistry to all students and other members of UK academic institutions.^29^ This online platform currently provides access to state-of-the-art chemistry databases and tools for the benefit of the chemical research community, with a data repository for UK chemical research data also under development. Ultimately, the development of a data repository could facilitate “forward-looking” deposition of published PhD theses. In other words, it will enable future PhD students or their university to deposit primary and (critically) metadata under embargo, allowing the work that has been done to be discoverable in a more controlled and timely manner.

Recognising that open access to research data carries a significant cost, thought also needs to be given to the long-term sustainability of a database of discoverable thesis experimental details. In direct parallel to the consideration of open data within the HEI sector, more Early Discovery collaborative agreements are being put in place between large pharmaceutical companies and partners, where the shared goals of the partners are to explore a greater diversity of compounds against diverse biological targets. This in turn creates the opportunity for an NCC to borrow from the concepts that have already been developed in thinking more broadly about how the metadata around compound ownership and origin impacts their ability to use information and maintain an audit trail.^30^ Open innovation partnerships encompass many different models^31^ and a searchable thesis collection should also develop in ways that aim to maximise researcher impact through increased collaboration, technology transfer and commercialisation whilst simultaneously lowering barriers to collaboration and licensing through reduction in the administrative thicket of patents, CDAs and MTAs.^32^,^33^

The question of physical samples relating to the pilot dataset was outside the scope of this project, but in the longer term the broader chemistry community (universities, end-users, CROs, funders) has an opportunity to engage around the case to translate the tangible *in silico* database to a physical collection and this should recognise the legacy and forward-looking components of an *in silico*-focussed activity.

## Conclusions

The National Compound Collection pilot study set out to achieve a series of key goals to demonstrate the level of “added value” around under-exploited chemical structure data that is available using published PhD theses as an “open access” (published and openly-available) resource.

We believe that this pilot study, in achieving the tasks we set ourselves, has demonstrated clearly that PhD theses, independent of the primary chemical literature, provide a highly valuable source of new chemical structure information. A thesis, as a published document, offers access to a quality controlled experimental procedure and by linking ChemSpider to the thesis detail, the postgraduate student author (and copyright holder) is credited more fully for the work that they have done.

We recognise where there remains work to be done; identifying those “unknown unknowns” was also a function of the pilot. Copyright issues may also link to any future use of the data collection and there is a clear need to coordinate the ways in which universities and the British Library convert paper-based theses to a usable and readily searchable electronic resource that harnesses the “added value” that is available.

We used a legacy (*i.e.* already published) set of theses and see real value in further legacy mining as well as engaging with the broader (and beyond UK) academic community to harvest new theses into a data collection as they are produced and cleared for publication.^34^

As has been noted elsewhere, we are moving towards a more open world, in which organisations need to collaborate in order to thrive.^35^,^36^ The old, linear paradigm where each player's position was clearly defined has evolved into a dynamic network of non-traditional partnerships in which compounds, data, expertise and knowledge are shared.^37^,^38^ The development of a repository that makes a significant volume of publicly-funded research openly-available will become increasingly valuable in an environment where the roles of industry, academia, charities and research funders in innovation are increasingly overlapping.

## Supplementary Material

Supplementary information
