# Supplementary material for: The creation and characterisation of a National Compound Collection: the Royal Society of Chemistry pilot
Source: Chem Sci. 2016 Feb 23;7(6):3869–78. doi: 10.1039/c6sc00264a (PMC6013800; doi:10.1039/c6sc00264a)
Supplement: Supplementary file 1 [file SC-007-C6SC00264A-s001.zip › Acknowledgements.docx]

www.rsc.org/

Detailed Acknowledgement of Contributions

Acknowledgements

We thank University of Bristol (UoB), including the Research and Enterprise Development group (Sue Sundstrom and Neil Bradshaw) and the Elizabeth Blackwell Institute (Professor Jeremy Tavare) for initial seed corn funding, University of Huddersfield (UoH) and the British Library (BL) for their active participation and provision of staff time, SimBioSys Inc for free trial access to CLiDE software, and the Royal Society of Chemistry (RSC) for funding of this pilot study.

A large number of other people and organisations contributed to and advised on many aspects of this project and informed this report. We recognise the key roles played by: our “data collectors” and those in their institutions who supported them by engaging colleagues, providing both advice to the data collectors and copies of theses, as well as those who organised and implemented the numerous contractual arrangements involved; Richard Sessions who coordinated the in silico screening activity; Steve Garland who coordinated the multicomponent activity around assessment/comparison of the in silico collection by a number of external partners; Tony Williams for leading the ChemSpider activity; Sara Gould for coordinating the activities associated with the British Library.

The universities and the people within them who supported this project from the outset: Bath, Bradford, Cambridge, Cardiff, Dundee, Glasgow, Huddersfield, Imperial College, Nottingham, Leeds, Leicester, Loughborough, Strathclyde and UCL; and Hull and St Andrews for additional help with e-theses.

We had very generous and constructive advice from contributors drawn from a large number of other groupings such as Research Councils (EPSRC, BBSRC and MRC), Wellcome Trust and BIS, a number of pharma (GSK, AZ, Lilly, Domainex, Argenta, Evotech, Cresset, IMI European Lead Factory) and agrochemical (Syngenta) companies, CROs (Peakdale, Albany Molecular, Sygnature, SORD) and not for profit and related organisations and consortia such as ICR (Julian Blagg), MRCT (Andy Merritt), CRT, European Screening Port, EMBL-EBI, and Stevenage Bioscience Catalyst (Stan Roberts and Martino Picardo), Dial-a Molecule (Richard Whitby), 3D Fragments, CIKTN, EU OpenScreen.

The cooperation of French colleagues around the French-based compound collection (Chimiotheque Nationale) was much appreciated, as is the support provided by those bioscience colleagues, within Bristol and externally, who contributed their protein structures to the in silico screening aspect.

We particularly wish to acknowledge the key roles played the following people in delivering the various components of this pilot study: Amaurys Avila Ibarra (UoB), Sylviane Boucharens (BioAscent Discovery Ltd), Paul Brennan (SGC), Alan Brown (Pfizer), Luis Castro (UCB), David Clark (CRL Discovery Services), Sara Gould (BL), Debra Hiom (UoB), David James (RSC), Phil Jones (IMI-funded European Lead Factory), Gregor MacDonald (J&J), Brian Marsden (SGC), Alex Mayweg (Roche), David Pryde (Pfizer), Dave Shepard (CRL Discovery Services), Gus Slater (UoB), Richard Sessions (UoB), Valery Tkachenko (RSC), Magnus Walter (Lilly) and Tony Williams (RSC).
